# Impact of medical student origins on the likelihood of ultimately practicing in areas of low vs high socio-economic status

**DOI:** 10.1186/s12909-016-0842-7

**Published:** 2017-01-05

**Authors:** Ian B. Puddey, Denese E. Playford, Annette Mercer

**Affiliations:** 1 School of Medicine and Pharmacology, Faculty of Medicine, Dentistry and Health Sciences, University of Western Australia, Level 4 RPH MRF Building, Rear 50 Murray St, Perth, WA 6000 Australia; 2 School of Primary, Aboriginal and Rural Health Care, Faculty of Medicine, Dentistry and Health Sciences, University of Western Australia, 35 Stirling Hwy, Crawley, WA 6009 Australia; 3 Faculty Office, Faculty of Medicine, Dentistry and Health Sciences, University of Western Australia, 35 Stirling Hwy, Crawley, WA 6009 Australia

**Keywords:** Medical workforce, Medically underserved areas, Medical students, Widening access, Socio-economic status

## Abstract

**Introduction:**

Medical schools are in general over-represented by students from high socio-economic status backgrounds. The University of Western Australia Medical School has been progressively widening the participation of students from a broader spectrum of the community both through expanded selection criteria and quota-based approaches for students of rural, indigenous and other socio-educationally disadvantaged backgrounds. We proposed that medical students entering medical school from such backgrounds would ultimately be more likely to practice in areas of increased socio-economic disadvantage.

**Methods:**

The current practice address of 2829 medical students who commenced practice from 1980 to 2011 was ascertained from the Australian Health Practitioner Regulation Agency (AHPRA) Database. Logistic regression was utilised to determine the predictors of the likelihood of the current practice address being in the lower 8 socio-economic deciles versus the top 2 socio-economic deciles.

**Results:**

Those who were categorised in the lower 8 socio-economic deciles at entry to medical school had increased odds of a current practice address in the lower 8 socio-economic deciles 5 or more years after graduation (OR 2.05, 95% CI 1.72, 2.45, *P* < 0.001). Other positive univariate predictors included age at medical degree completion (for those 25 years or older vs those 24 years or younger OR 1.53, 95% CI 1.27, 1.84, *P* < 0.001), being female (OR 1.26, 95% CI 1.07, 1.48, *P* = 0.005) and having a general practice versus specialist qualification (OR 4.16, 95% CI 3.33, 5.19, P < 0.001). Negative predictors included having attended an independent school vs a government school (OR 0.77, 95% CI 0.64, 0.92, *P* < 0.001) or being originally from overseas vs being born in Oceania (OR 0.80, 95% CI 0.67, 0.96, *P* = 0.017). After adjustment for potential confounders in multivariate logistic regression, those in the lower 8 socio-economic deciles at entry to medical school still had increased odds of having a current practice address in the lower 8 socio-economic deciles (OR 1.63, 95% CI 1.34, 1.99, *P* < 0.001).

**Conclusion:**

Widening participation in medical school to students from more diverse socio-educational backgrounds is likely to increase the distribution of the medical workforce to ultimate service across areas representative of a broader socio-economic spectrum.

**Electronic supplementary material:**

The online version of this article (doi:10.1186/s12909-016-0842-7) contains supplementary material, which is available to authorized users.

## Background

The famous Prussian pathologist Rudolf Virchow, also a prominent figure in the field of social medicine, once stated that “The physicians are the natural attorneys of the poor, and the social problems should largely be solved by them.” [1]. A continuing social problem however, in Australia and globally [2], is maldistribution of the medical workforce, with fewer graduating physicians electing to serve in traditionally underserved rural and outer urban communities despite high population doctor to patient ratios. Maldistribution of the medical workforce is an almost universal problem, occurring in countries large and small, rich and poor [2]. In particular, the generalist primary care workforce for underserved communities is diminishing in Australia with graduates increasingly choosing to train in the specialties and sub-specialties [3].

The definition of underserved populations clearly includes rural health workforce shortages, but also healthcare for those living in socially disadvantaged metropolitan areas within cities. When modelling of patient service provision in Australia has been carried out on the basis of socio-economic indices (the Socio-Economic Indices for Areas (SEIFA) Index of Relative Social Disadvantage score) a complex relationship in socially disadvantaged metropolitan areas has emerged [4]. Higher utilisation of general practitioner services was seen for people living in low vs high socio-economic areas and interpreted as being consistent with those from lower socio-economic areas having poorer health. Yet when very low SEIFA index areas were analysed in isolation, utilisation of general practitioner services was quite low, an observation thought to reflect issues of affordability as well as lack of access. When the catchment areas of every general practice surgery in the capital city of Perth, Western Australia, were geo-coded into quartiles of social disadvantage and the equity of delivery of medical services assessed, again complex patterns emerged [5]. Although doctor-hours of service provision were greater in the most disadvantaged areas, there was a higher throughput of patients, shorter consultation times and a lesser likelihood of being able to be provided with a same day appointment. In addition there was poorer spatial access to surgeries, evening services were less likely to be provided and practices were also less able to provide a choice of gender of practitioner. Similarly, Roeger et al. [6] utilised the SEIFA Index of Relative Social Advantage and Disadvantage score to examine the equity of access to general practitioners in the capital city, Adelaide, South Australia, and found that residents in the outer suburbs and in those with lower socio-economic status (SES) appeared to be the most disadvantaged. They demonstrated an approximate linear relationship between SES and the mean population to general practitioner ratio, with the poorest areas increasingly underserved.

In one of the first studies to examine factors that determine generalist physicians’ care of the underserved, 1704 graduates from a US medical school were followed up for approximately 10 year after graduation [7]. The significant predictors of those currently providing substantial care to underserved populations included being a member of an underserved minority, having previously participated in the National Health Service Corps, having a strong interest in practicing in an underserved area before medical school, and having grown up in an underserved area. Those who grew up in an underserved area had a 1.6 fold increase in the odds of currently providing substantial care to an underserved population. One of the limitations of that study was that it failed to differentiate between physicians who grew up or currently practiced in underserved rural areas vs those in underserved areas in the city. In 2009 Wayne et al. [8] followed up 244 graduates from their medical school’s class of 1997 to ascertain if they had practiced in a medically underserved community in the past year. Rural background, older age and being a member of an underrepresented minority were the independent predictors of current practice in an underserved community. Odom Walker et al. [9] stratified physicians from Los Angeles County on the basis of practice location (underserved vs non-underserved area) and conducted interviews to establish themes influencing the decision to practice in such areas. Self-identity including socioeconomic and geographic background of the physician was one of the driving forces identified together with a sense of responsibility and commitment to the community as well as the opportunity for personal development. Community influence with a strong attachment to original home community has also been invoked as a determinant of graduates ultimately practicing in an underserved community [10].

In medical schools in Australia and elsewhere there has been an increasing awareness of greater social accountability in medical education including a desire to graduate more medical practitioners who will choose to practice in underserved communities [11]. An international study of 6 geographically dispersed medical schools looked at students’ intentions to practice in an underserved area after graduation [12]. The schools were united by a common social accountability mandate that led to the selection of relatively higher proportions of students from underserved populations. High proportions of students from all 6 schools indicated an intention to practice in underserved communities. However, whether such intention ultimately translates into actual practice in an underserved community remains a matter of contention. Longitudinal cohort studies in medical schools in Australia that have attempted to answer this question, in terms of years of follow-up, have been of relatively short duration and have largely focused on predictors of rural practice alone [13–17]. In these studies, a rural background and prolonged immersion in a rural clinical training environment have been the major factors influencing increased likelihood of rural practice.

For more than 2 decades The UWA Medical School has been progressively widening the participation of students from a broader spectrum of the community, both through expanded selection criteria and quota-based approaches for students of rural, indigenous and other socio-educationally disadvantaged backgrounds. This has resulted in an increase in the number of students recruited from the lowest 8 socioeconomic deciles and a decrease in those from the upper 2 deciles. The major motivation for these initiatives has been the principle of social equity with our medical school, in common with most others [18, 19], heavily over-represented by students from high SES backgrounds. In the present study, we have now asked the question as to whether there might be an additional benefit from having widened medical student access, with the hypothesis that those entering medical school from relatively socio-economically disadvantaged backgrounds will ultimately be more likely to practice in areas of increased socio-economic disadvantage.

## Methods

All domestic students entering the UWA medical school from 1980 and who had completed their degree by 2011 were considered for study inclusion. Age, gender, type of school attended (government (publicly funded) or independent (fee paying)) and self-reported indigenous background (Aboriginal or Torres Strait islander) - were recorded at entry to medical school.

Entry into the 6 year undergraduate MBBS course was either by a standard pathway, with direct entry from secondary school, or a non-standard pathway, for students who had already completed some tertiary study in another course. Selection into either pathway from 1980 until 1998 was on the basis of prior academic performance alone as assessed by the Australian Tertiary Admissions Rank (ATAR) for standard entry students or grade point average (GPA) for non-standard students. From 1999 onwards selection factors were broadened to also include a structured interview and an aptitude test – the Undergraduate Medicine and Health Sciences Admission Test (UMAT) [20]. From 2005, a further graduate entry pathway commenced with subjects who had already completed a tertiary degree enrolled into a 6 month bridging course before entering level 3 of the 6 year undergraduate MBBS. They were also selected using the broadened criteria of prior academic performance (GPA), a structured interview and an aptitude test – the Graduate Australian Medical School Admission Test (GAMSAT) [21]. For all subjects a dichotomous dummy variable (Selection factors) was constructed, with those selected using ATAR or GPA alone designated zero and those selected using ATAR or GPA, interview score and either UMAT or GAMSAT score designated as unity.

For students admitted from 1980 to 1998 no specific process was in place to define all rural background students and so for the purposes of this study they have been defined as rural if they either had a rural correspondence address at entry to the course and/or completed secondary school in an area defined as Australian Standard Geographical Classification - Remoteness Area 2 to 5 [22]. For those who entered from 1999 to 2007 applicants were considered rural if they had lived in a rural area of Western Australia for a minimum of two years and, during that period, completed year 12 at a rural secondary school – “rural” being defined as a distance of >75kms from the Perth Central Business District.

Region of origin was determined from country of origin according to major regional groups as outlined in the Australian Standard Classification of Countries for Social Statistics [23]. Given the relatively small numbers of students in some groups they were collapsed into 5 groups for analysis - those from Oceania (Australia, New Zealand, Papua New Guinea and proximate Pacific islands), UK and Ireland, NE and SE Asia, Southern Asia (India, Pakistan, Sri Lanka and Bangladesh) and Other.

From 2001 and 2004, respectively, 2 schemes were developed which obligate students to a period of return of service in either a regional, rural or remote area (the Medical Rural Bonded Scholarship Scheme – MRBS) or a District of Workforce Shortage (the Bonded Medical Places Scheme – BMP). The MRBS requires individuals to work in a regional, rural or remote area for up to 6 years once they qualify as a medical specialist or a general practitioner. Following attainment of Fellowship of a specialist college, students accepting a BMP agree to work in a District of Workforce Shortage (DWS) of their choice for a period equal to the length of their medical degree. A DWS is a geographical area in which the local population has less access to Medicare subsidised medical services when compared to the national average. This period can be scaled down depending on the relative remoteness of the area in which they ultimately choose to work. All students enrolled in either of these schemes were identified and dummy predictor variables constructed to allow for any potential influence of such schemes on the major outcome variable.

As a socioeconomic indicator, the correspondence postcode at entry for each student was linked to the Index of Relative Socioeconomic Advantage and Disadvantage (IRSAD) score from the Australian 2006 census SEIFA [24]. Therefore the final analysis was confined to only Australian citizens and permanent residents with fee paying International students, whose previous addresses had been overseas, excluded. The construct for SEIFA codes, and the caveats in relation to their use as socio-economic indicators, have previously been described [25]. A dummy variable was constructed which dichotomised the cohort into the top 2 deciles for IRSAD score at medical school entry vs the bottom 8 deciles.

In April 2016 the current practice address of all graduates was ascertained through utilisation of the Australian Health Practitioner Regulation Agency (AHPRA) database. The postcode of the current practice address was utilised to generate an IRSAD score which was once again dichotomised into the top 2 deciles vs the bottom 8 deciles. Current type of registration (general registration, general practice or specialist practice) was also ascertained from the AHPRA database.

A searchable map [26] was utilised to further dichotomise those whose current practice was located in a major city as either inner or outer metropolitan with outer metropolitan defined as “the part of the State capital city Statistical Division (using the 2001 Australian Standard Geographic Classification definition) that lies outside the 1991 Urban Centre area of the capital city” [27].

### Statistics

Data were analysed using IBM SPSS Statistics Release 21.0.0. All values are presented as mean ± SD. Univariate comparisons of IRSAD decile 1–8 vs IRSAD decile 9–10 based on address at medical school entry were made using the *χ* ^2^ test. Univariate analysis of predictors of ultimately practicing in an IRSAD decile 1–8 practice address vs IRSAD decile 9–10, or of practicing in an outer vs inner metropolitan region of a capital city, utilised logistic regression. A final multivariate model was constructed for the major outcome variable of current decile of practice (1 to 8 vs 9–10), using logistic regression with block entry at step 1 of socio-demographic variables with potential predictive value, followed by IRSAD decile at medical school entry (1 to 8 vs 9–10) at step 2. There were 308 students with missing data for country of origin and 587 students with missing data for type of secondary school. All other variables had complete data sets. For the multivariate logistic regression model, therefore, those with missing data were conservatively designated as being born in Oceania or having attended a public school, respectively.

## Results

Overall, 3149 graduates were initially eligible for inclusion. Subsequently excluded from the study were 19 graduates with either an international or no local correspondence address recorded at medical school entry, 194 either not currently listed on the AHPRA 2016 database, suspended in 2016 or failed to re-register in 2016, 7 who were deceased and 100 who were either overseas or registered as currently non-practicing leaving 2829 graduates in the final analysis (89.8% of the total cohort).

The final cohort had a mean age of 24.6 ± 3.5 years at completion of the course. Approximately 48% were female (*N* = 1353) and 52% were male (*N* = 1476). There were 57% from an independent school background (*N* = 1285) and 43% from a government school background (*N* = 957). This is against a background Western Australian ratio of 41% attending independent schools and 59% government schools from 2007 to 2010 [28]. There were 93% of urban origin (*N* = 2638) and 7% of rural origin (*N* = 191) while more broadly in Western Australia approximately 26% of the total population live rurally [28]. There were 66% who were born in Oceania (*N* = 1651) while 34% had migrated to Australia from other countries (*N* = 870). The cohort had been in the workforce for 16.5 ± 8.0 year (range 5–33 years). Approximately 27% were from the lower 8 IRSAD deciles at medical school entry (*N* = 749) and 73% from the upper 2 IRSAD deciles (*N* = 2080) while for those who lived in capital cities, 92.5% (*N* = 2377) were from the inner metropolitan area and 7.5% (*N* = 193) were from the outer metropolitan area.

Affirmative action policies for students of rural origin (initiated in 1993), indigenous origin (initiated in 1996) or those with other socio-educational disadvantage (UWay – also initiated in 1996) with subsequent establishment of specific quotas for these groups resulted in 6% of the cohort being admitted via quota-based pathways (*N* = 167) from 1980 to 2007, 2.5% (*N* = 44) before 1999 and 11.7% (*N* = 123) after. A bonded medical place had been accepted by 65 students (2.3%) and a medical rural bonded scholarship by 48 students (1.7%). With respect to selection, 59.6% were admitted prior to 1999 on the basis of academic merit alone (*N* = 1685) while 40.4% were admitted after 1999 when widened selection criteria had been introduced (*N* = 1144). Since the introduction of broadened selection criteria, quota-based entry programs for rural, indigenous and socio-educationally disadvantaged students and the graduate entry stream, the numbers of students originating from the top 2 IRSAD deciles has fallen from 78.5 to 65.2% with a commensurate increase in those in the lower 8 deciles from 21.5 to 34.8% (*χ* ^2^ = 59.7, *P* < 0.001) (Table 1).

**Table 1:** Socio-economic status (as measured by IRSAD decile) pre and post introduction of expanded selection criteria in 1999, together with expansion of quota-based approaches for students of rural, indigenous and socio-educationally disadvantaged backgrounds (*χ* ^2^ = 94.3, *P* < 0.001)

	IRSAD Decile at Entry to Medical School										
	1	2	3	4	5	6	7	8	9	10	Total
Pre 1999	3 (0.2%)	13 (0.7%)	13 (0.7%)	16 (0.9%)	43 (2.4%)	63 (3.6%)	125 (7.0%)	106 (6.0%)	378 (21.3%)	1014 (57.2%)	1774 (100%)
Post 1999	2 (0.2%)	10 (0.9%)	21 (2.0%)	21 (2.0%)	70 (6.6%)	78 (7.4%)	62 (5.9%)	103 (9.8%)	193 (18.3%)	495 (46.9%)	1055 (100%)
Total	5	23	34	37	113	141	187	208	571	1509	2829

Selection factors and socio-demographic factors by IRSAD decile of address at medical school entry to the course are listed in Table 2. Selection via the widened group of selection factors and/or a special entry pathway were both associated with a significantly higher number of students being within the lower 8 deciles. Students in the lower 8 deciles were also significantly more likely to be older at completion of the course. They were significantly less likely to have attended independent (fee paying) rather than government (publicly funded) schools and less likely to have originally been of NE or SE Asian origin. They were more likely to have accepted a MRBS or a BMP.

**Table 2:** Selection and socio-demographic factors by high versus low IRSAD decile for address at medical school entry (percentages are listed within each factor category)

	*N*	IRSAD Decile at Medical School Entry 1-8	*N*	IRSAD Decile at Medical School Entry 9-10	*P*-Value (*χ* ^2^ test)
Selection factors					**<0.001**
ATAR or GPA alone	362	48.3%	1323	67.0%	
ATAR or GPA, UMAT or GAMSAT and Interview Score	387	51.7%	757	36.4%	
Quota-based entry pathway					**<0.001**
No quota-based entry	606	80.9%	2056	98.8%	
Quota-based entry	143	19.1%	24	1.2%	
Medical Rural Bonded Scholarship					**<0.001**
No medical rural bonded Scholarship	723	96.5%	2058	98.9%	
Medical rural bonded scholarship	26	3.5%	22	1.1%	
Bonded Medical Place					**0.032**
No bonded medical place	724	96.7%	2040	98.1%	
Bonded medical place	25	3.3%	40	1.9%	
Age at completion					**<0.001**
Up to 24 years	519	69.3%	1643	79.0%	
25 years and older	230	30.7%	437	21.0%	
Sex					0.115
Female	377	50.3%	976	46.9%	
Male	372	49.7%	1104	53.1%	
Secondary school					**<0.001**
Government	294	51.4%	663	39.7%	
Independent	278	48.6%	1007	60.3%	
Country of Origin					**<0.001**
Oceania	442	64.1%	1044	57.0%	
UK and Ireland	37	5.4%	128	7.0%	
NE and SE Asia	101	14.7%	414	22.6%	
Southern Asia	29	4.2%	67	3.7%	
Other	80	11.6%	179	9.8%	

Significant *P* values are in bold-faced type

The IRSAD decile of the current practice address together with the current type of medical registration are listed in Table 3 for all graduates by IRSAD decile of address at medical school entry. After graduation, those from the lower 8 IRSAD deciles were significantly more likely to have a current practice address in the lower 8 IRSAD deciles (OR 2.05, 95% CI 1.72, 2.45, *P* < 0.001). They were also approximately twice as likely to be either generally registered (OR 2.18, 95% CI 1.79, 2.66, *P* < 0.001) or in general practice rather than in specialist practice (OR 1.90, 95% CI 1.51, 2.38, *P* < 0.001).

**Table 3:** Registration type and IRSAD decile of practice address by high versus low IRSAD decile for address at medical school entry (percentages are listed within each factor category)

	*N*	IRSAD Decile at Medical School Entry 1-8	N	IRSAD Decile at Medical School Entry 9-10	*P*-Value (*χ* ^2^ test)
Registration Type					**<0.001**
Specialist Practice	199	26.6%	892	42.9%	
General Registration	361	48.2%	741	35.6%	
General Practice	189	25.2%	447	21.5%	
IRSAD Decile of Practice Address					**<0.001**
Deciles 1-2	15	2.0%	18	0.9%	
Deciles 3-4	26	3.5%	45	2.2%	
Deciles 5-6	145	19.4%	226	10.9%	
Deciles 7-8	118	15.8%	231	11.1%	
Deciles 9-10	445	59.4%	1560	75.0%	

Significant *P* values are in bold-faced type

Results of univariate logistic regression analysis of potential predictors of a low (1–8) vs high (9–10) IRSAD decile for current practice address are listed in Table 4. The odds of being in the lower 8 deciles were significantly increased in those who entered via a quota-based entry program but not for those selected for entry by the widened group of selection factors. Those in the lower 8 deciles also had a significant increase in the odds of having accepted a MRBS but not a BMP. Both older age at graduation and being female increased the odds of being in a practice address in the lower 8 deciles. A reduction in the odds was seen for those from independent vs government schools and in students originally of NE and SE Asian origin while a significant increase in the odds was seen in those with general registration and those registered as general practitioners vs other specialties.

**Table 4:** Univariate predictors of graduates currently in practice in low (1^st^ to 8^th^) versus high (9^th^ to 10^th^) IRSAD decile practice address

	Number (%) currently in practice address with IRSAD decile 1-8	Odds ratio (Logistic regression)	*P*
Selection factors			
ATAR or GPA alone	471/1685 (28.0%)	1.0	
ATAR or GPA, UMAT or GAMSAT and Interview score	353/1144 (30.9%)	1.15 (0.98, 1.36)	0.097
Quota-based entry pathway			
No quota-based entry	741/2662 (27.8%)	1.0	
Quota-based entry	83/167 (49.7%)	2.56 (1.87, 3.51)	**<0.001**
Medical Rural Bonded Scholarship			
No Medical rural bonded scholarship	796/2781 (28.6%)	1.0	
Medical rural bonded scholarship	28/48 (58.3%)	3.49 (1.96, 6.23)	**<0.001**
Bonded Medical Place			
No bonded medical place	803/2764 (29.1%)	1.0	
Bonded medical place	21/65 (32.3%)	1.17 (0.69, 1.97)	0.568
Age at completion			
Up to 24 years	583/2162 (27.0%)	1.0	
25 years and older	241/667 (36.1%)	1.53 (1.27, 1.84)	**<0.001**
Sex			
Male	396/1476 (26.8%)	1.0	
Female	428/1353 (31.6%)	1.26 (1.07, 1.48)	**0.005**
Secondary school			
Government	289/957 (30.2%)	1.0	
Independent	320/1285 (24.9%)	0.77 (0.64, 0.92)	**0.006**
Country of Origin			
Oceania	448/1486 (30.1%)	1.0	
UK and Ireland	53/165 (32.1%)	1.10 (0.78, 1.55)	0.605
NE and SE Asia	124/515 (24.2%)	0.74 (0.59, 0.93)	**0.010**
Southern Asia	27/96 (28.1%)	0.91 (0.57, 1.43)	0.672
Other	73/259 (28.2%)	0.91 (0.68, 1.22)	0.520
Registration Type			
Specialist Practice	185/1091 (17.0%)	1.0	
General Registration	347/1102 (31.5%)	2.25 (1.84, 2.76)	**<0.001**
General Practice	292/636 (45.9%)	4.16 (3.33, 5.19)	**<0.001**
IRSAD Decile of Address at Entry			
Deciles 1-2	18/28 (64.3%)	5.40 (2.48, 11.77)	**<0.001**
Deciles 3-4	26/71 (36.6%)	1.73 (1.06, 2.84)	**0.029**
Deciles 5-6	119/254 (46.9%)	2.64 (2.03, 3.45)	**<0.001**
Deciles 7-8	141/396 (35.6%)	1.66 (1.32, 2.08)	**<0.001**
Deciles 9-10	520/2080 (25.0%)	1.0	

Significant *P* values are in bold-faced type

A multivariate logistic regression model was constructed with being in the lower (1–8) vs higher (9–10) deciles for current practice address as the dependent variable (Table 5). Being in the lower 8 IRSAD deciles at entry to medical school remained an independent predictor of being in a current practice address that was in the lower 8 IRSAD deciles (OR 1.63, 95% CI 1.34, 1.99, *P* < 0.001). Entry to the course through a special entry pathway, having accepted a MRBS, higher age at completion of the course, and type of current registration (generally registered or general practice) each also significantly increased the odds of being in a current practice address that was in the lower 8 IRSAD deciles, while having attended an independent school decreased the odds.

**Table 5:** Multivariate logistic regression with low (1^st^ to 8^th^) vs high (9^th^ to 10^th^) IRSAD decile of current practice as the dependent variable and selection and socio-demographic factors as the predictor variables (*N* = 2829) (Nagelkerke R Square = 0.12)

Predictor Variable	B	S.E.	*P* Value	Odds Ratio (95% CI)
Quota-based entry pathway				
No quota-based entry				1.0
Quota-based entry	0.444	0.182	**0.015**	1.56 (1.09, 2.23)
Selection factors				
ATAR or GPA alone				1.0
ATAR or GPA, UMAT or GAMSAT and Interview score	−0.124	0.125	0.318	0.88 (0.69, 1.13)
Medical Rural Bonded Scholarship				
No Medical Rural Bonded Scholarship				1.0
Medical Rural Bonded Scholarship	0.860	0.313	**0.006**	2.36 (1.28, 4.37)
Bonded Medical Place				
No bonded medical place				1.0
Bonded medical place	0.007	0.281	0.980	1.01 (0.58, 1.75)
Age at Completion				
24 years or younger				1.0
25 years or older	0.233	0.104	**0.025**	1.26 (1.03, 1.55)
Sex				
Male				1.0
Female	0.029	0.089	0.749	1.03 (0.86, 1.23)
School Type				
Government				1.0
Independent	−0.207	0.092	**0.024**	0.81 (0.68, 0.97)
Country of Origin				
Oceania				1.0
Other	−0.179	0.094	0.055	0.84 (0.70, 1.00)
Registration Type				
Specialist				1.0
General registration	0.687	0.136	**<0.001**	1.99 (1.52, 2.60)
General Practice	1.326	0.116	**<0.001**	3.77 (3.00, 4.73)
IRSAD Decile of Address at Entry				
Deciles 1-8	0.491	0.100	**<0.001**	1.63 (1.34, 1.99)
Deciles 9-10				1.0

Significant *P* values are in bold-face type

A rural postcode is more likely to be associated with a lower IRSAD decile and we have previously demonstrated that a rural background increases the odds of subsequent rural practice [7]. Therefore a further multivariate regression model (Additional file 1: Table S1) was constructed for urban background students alone (*N* = 2638). This saw similar results for the odds of being in a current practice address in the lower 8 deciles for those who had been in the lower 8 IRSAD deciles at entry to medical school (OR 1.53, 95% CI 1.25, 1.88, *P* < 0.001).

Furthermore, when those whose current practice was in a capital city were analysed alone (*N* = 2473), in a univariate logistic regression analysis the odds of practicing in an outer vs inner metropolitan area were significantly increased in those who had been in the lower 8 IRSAD deciles at entry to medical school (OR 1.77, 95% CI 1.40, 2.25, *P* < 0.001). When the IRSAD deciles were broken down further into 5 categories a linear trend was evident in the odds ratio of current medical practice being in an outer vs inner metropolitan area (Table 6). The odds of being in an outer vs inner metropolitan practice was significantly increased in those who entered via a quota-based entry program but not for those selected for entry by the widened group of selection factors or those who accepted either a BMP or a MRBS. Age at graduation, gender and type of secondary school were not significant predictors while reduced odds were seen for those students originally of NE and SE Asian origin (Table 6). There was a significant increase in the odds of outer metropolitan practice in both those with general registration and those registered as general practitioners vs other specialty practice. In a multivariate model that included all relevant univariate predictors, the odds ratio of current medical practice being in an outer vs inner metropolitan area still remained highly significant (OR 1.52, 95% CI 1.17, 1.97, *P* = 0.001) (Additional file 1: Table S2).

**Table 6:** Univariate predictors of capital city based graduates currently in practice in an outer metropolitan versus inner metropolitan practice address (*N* = 2473)

	Number (%) currently in practice in outer metropolitan area	Odds ratio (Logistic regression)	*P*
Selection factors			
ATAR or GPA alone	218/1488 (14.7%)	1.0	
ATAR or GPA, UMAT or GAMSAT and Interview score	153/985 (15.5%)	1.07 (0.86, 1.34)	0.547
Quota-based entry pathway			
No quota-based entry	340/2354 (14.4%)	1.0	
Quota-based entry	31/119 (26.1%)	2.09 (1.36, 3.19)	**0.001**
Medical Rural Bonded Scholarship			
No Medical rural bonded scholarship	363/2444 (14.9%)	1.0	
Medical rural bonded scholarship	8/29 (27.6%)	2.18 (0.96, 4.97)	0.063
Bonded Medical Place			
No bonded medical place	359/2419 (14.8%)	1.0	
Bonded medical place	12/54 (22.2%)	1.64 (0.86, 3.14)	0.137
Age at completion			
Up to 24 years	276/1923 (14.4%)	1.0	
25 years and older	95/550 (17.3%)	1.25 (0.97, 1.61)	0.091
Sex			
Male	187/1175 (15.9%)	1.0	
Female	184/1298 (14.2%)	1.15 (0.92, 1.43)	0.227
Secondary school			
Government	136/839 (16.2%)	1.0	
Independent	154/1154 (13.3%)	0.80 (0.62, 1.02)	0.074
Country of Origin			
Oceania	210/1268 (16.6%)	1.0	
UK and Ireland	24/142 (16.9%)	1.03 (0.65, 1.63)	0.918
NE and SE Asia	57/484 (11.8%)	0.67 (0.49, 0.92)	**0.011**
Southern Asia	12/82 (14.6%)	0.86 (0.46, 1.62)	0.599
Other	34/226 (15.0%)	0.89 (0.60, 1.32)	0.686
Registration Type			
Specialist Practice	105/1033 (10.2%)	1.0	
General Registration	160/951 (16.8%)	1.79 (1.37, 2.33)	**<0.001**
General Practice	106/489 (21.7%)	2.45 (1.82, 3.29)	**<0.001**
IRSAD Decile of Address at Entry			
Deciles 1-2	9/20 (45.0%)	5.45 (2.24, 13.28)	**<0.001**
Deciles 3-4	20/62 (32.3%)	3.17 (1.83, 5.49)	**<0.001**
Deciles 5-6	39/187 (20.9%)	1.76 (1.20, 2.56)	**0.004**
Deciles 7-8	59/335 (17.6%)	1.42 (1.04, 1.94)	**0.026**
Deciles 9-10	244/1869 (13.1%)	1.0	

Significant *P* values are in bold-faced type

## Discussion

One of the goals of the revised selection processes at our medical school was to increase the diversity in the student cohorts. In an earlier report [28], where we included only standard entrants from secondary school and relied on a socioeconomic index of each student’s secondary school as the marker of socio-educational disadvantage, no increase in diversity was seen. In the current expanded study where each student’s correspondence address at entry to medical school was utilised as a surrogate for determination of socio-educational advantage or disadvantage, it has become clear that both changing the selection criteria in 1999, together with expansion of the quota-based entry approaches for students of rural, indigenous and socio-educationally disadvantaged backgrounds, has been associated with greater representation from those from the lower 8 IRSAD deciles, the numbers of students increasing from 21.5 to 34.8%. Given an approximate doubling of the numbers of medical students recruited and completing their studies at UWA from 1999 to 2011, the increase in absolute terms was even greater (20 students who graduated pre-1999 vs 64 students post-1999 originated in the lower 8 deciles).

The findings of the present study therefore, where there was an independent 1.63 fold increase in the odds of such students currently practicing in an area of relatively lower socio-economic disadvantage, provide further support for the efficacy of approaches that aim to increase diversity amongst medical school entrants. Widening access to students from lower SES groups appears to enhance convergence between the demographic profiles of students at entry to medical school with currently unmet health workforce needs. This extends not just to rural health workforce participation, with the observation in the current study of a 1.52 fold increase in the odds of students from the lower 8 IRSAD deciles currently practicing in an outer vs inner metropolitan area. These estimates are of similar magnitude to the previously discussed study of Rabinowitz et al. [7] where there was a 1.6 fold increase in the odds of currently providing substantial care to an underserved population in those who had grown up in an underserved area.

Data from the UK indicates that only 15% of medical students come from the lowest socio-economic groups [19] while comparable data from the US indicates that less than one-quarter of medical students come from families in the bottom 3 quintiles of family income [18]. However, similar to Australia, in the US there has been a large scale expansion of medical student numbers. A comparison of graduates from the pre-expansion period 1999–2001 with those from 2009–2011 post-expansion [29] indicated that those medical schools with the most expansion over the decade have produced the highest proportion of physicians practicing in underserved and rural areas. Much of this may relate to the opportunities taken during expansion to target greater recruitment of students from minorities and from underserved areas. These opportunities continue with the recent 2015 US medical school enrolment survey indicating that 50-75% of schools either have implemented within the last 2 years or already have in place programs for the recruitment of students from disadvantaged backgrounds or from rural and/or underserved communities while more than 80% have already established programs or policies for admission of minority students [30].

The type of secondary school attended by our graduates gives further support to the hypothesis that those entering medical school from relatively socio-economically disadvantaged backgrounds would ultimately be more likely to practice in an area of increased socio-economic disadvantage. Entrants to our medical school who were from the upper 2 IRSAD deciles exhibited a 1.6 fold increase in the odds of having graduated from an independent (fee paying) secondary school vs a government (publicly funded) secondary school. However, these independent secondary school graduates were significantly less likely (19% decrease in the odds ratio) to be practicing in an area in the lowest 8 IRSAD deciles, even when socio-economic background was already included in the final multivariate model.

Admission through quota-based entry programs remained a significant predictor of current practice in an area of relative socio-economic disadvantage in the final multivariate analysis. In contrast the implementation of diversified selection factors for entry into medical school, with the addition of an interview and aptitude tests rather than selection on academic ability alone, did not remain a significant predictor. This result may reflect previous observations of unanticipated effects of SES on performance in aptitude tests for medical student entry. In Australia better performance in the Undergraduate Medicine and Health Sciences Admission Test has been linked to an increase in SES [25], while ethnicity, parental occupation and attendance at independent or grammar schools predicts better performance in the UK Clinical Aptitude Test [31]. In a Canadian study of entrants to 6 medical schools [32] the addition of a multiple mini-interview score and the score from the Medical College Admission Test failed to neutralise the diversity limiting effect of selection based on the grade point average from previous tertiary studies. In contrast, the implementation at UWA of selection via quota-based pathways for indigenous, rural and socio-educationally disadvantaged students was independently associated with a 1.56 fold increase in the odds of ultimately practicing in an area of relative socio-economic disadvantage. Therefore, the development of quota-based pathways into medical school for students from more diverse socio-educational backgrounds, rather than broadening of selection criteria alone, appears more likely to increase the distribution of the medical workforce to ultimate service across areas representative of a broader socio-economic spectrum.

Although the numbers are relatively small, not surprisingly, students who had accepted either a Bonded Medical Place or Medical Rural Bonded Scholarship, were more likely to have originated from the lower 8 IRSAD deciles at entry to medical school. At this time, however, only acceptance of an MRBS has translated into a current practice address in the lower 8 deciles. This is likely to be due to the later initiation of the BMP scheme with most students still in training and yet to qualify as a medical specialist or a general practitioner, at which point they will be begin their return of service obligation in an area of unmet need.

An important confounder that needs consideration is the choice of generalist vs specialist practice, with specialists more likely to be practicing in inner metropolitan areas in major cities while general practitioners and those generally registered are more likely to be practicing in outer metropolitan and underserved rural areas. In the current study, those from the lower 8 socioeconomic deciles at entry to medical school had an approximate 2-fold increase in the odds of being in generalist vs specialty practice. The question becomes whether their subsequent greater odds of practicing in an underserved area was because of a choice for generalist practice rather than a desire to practice in an underserved socially disadvantaged community. In a study of NZ domestic students the socio-economic decile of their secondary school was analysed against their intended choice of future medical career [33]. Those from high decile schools were twice as likely to indicate interest in internal medicine, surgery and their subspecialties while those from lower decile schools made fewer choices in relation to the range of options presented to them. This was interpreted as either lack of awareness of potential careers available in medicine or a more definite understanding of their intended career direction. However, in that study both the high and low decile students indicated similar interest in pursuing general practice.

It is possible that a reduced likelihood of pursuing specialty or subspecialty practice by those from lower socioeconomic backgrounds could be related to lower aspirations compared to their higher SES peers. In a broader Australian study in both primary and secondary school students the hypothesis that under-representation of low SES students in tertiary institutions and high prestige occupations might indeed reflect such a phenomenon was tested [34]. With respect to their measure of occupational certainty, they found weak associations between SES and making a tentative vs certain future job choice and a moderate association between SES and an unformed future job choice. There was also a weak relationship with occupational prestige with higher SES students expressing interest in slightly more prestigious occupations. This was most apparent when the career choice was as a medical practitioner. Further, lower SES students indicated higher motivation by the financial security of their ultimate occupation while high SES students were more likely to pursue their interests and passions. Similarly, in a US study undergraduate students who perceived themselves as having greater economic resources, social power and social prestige reported more certainty and self-efficacy with respect to their career decisions [35]. Whether such SES-linked attitudes eventually translate into choice by medical graduates to pursue a generalist vs specialist practice is speculative. Even after inclusion of choice of specialty in our final multivariate model, being in the lower socioeconomic deciles at entry to medical school still independently predicted current practice in a lower SES area.

Graduates who were migrants to Australia were less likely than those born in Oceania to currently be practicing in an area of relative social disadvantage. This was largely related to those from an NE or SE Asian background who exhibited a 1.8 fold increase in the odds of having come from the 2 highest IRSAD deciles at medical school entry. This mirrors the finding from an earlier study from UWA which focussed on predictors of rural practice and followed up 2 cohorts of students (those who commenced in 1984 and 1989) 4 years after graduation [36]. In that study rural versus metropolitan practice was equally positively predicted by either having ever lived in a rural area or by having been influenced to study medicine by a doctor, but negatively associated with having come from a non-European background. It is possible that those who have migrated to Australia have a diminished sense of an ongoing commitment to a community of origin and hence to underserved populations in general. Equally, practice in higher SES areas could be a surrogate for the fact that significantly more of these students choose to specialise or sub-specialise after graduation, ultimately ending up in inner urban practices.

Age was another potential confounder in our study with students in the lower 8 socioeconomic deciles at entry to medical school significantly older at completion of the medical program. Age at completion was also a positive independent predictor of ultimate practice location with those 24 years and older 26% more likely to be currently practicing in a low SES area. The duration of the program was identical for the 2 age groups studied and older age at completion was therefore largely due to an approximate 3 years age difference at program entry. This could reflect a necessary period of employment before a proportion of low SES students are in a financially viable position to enter medical school or it could reflect greater tentativeness before final commitment to the heavy demands of an intense medical program. Wayne et al. [8] noted a similar age relationship with those 25 years or older at entry to their medical program almost twice as likely to be currently in a medically underserved community. They suggested that older students may be good choices for medical schools interested in increasing the number of their graduates practicing in medically underserved communities. Gender was another prospective confounder in our study. Although there was no gender difference in terms of SES at entry to the medical school, there was a 26% increase in the odds that females would be found in practice in the lower 8 socioeconomic decile areas. This was no longer significant in multivariate analysis, perhaps reflecting the relative strength of specialty choice in the final model, females with a more than 2-fold increase in the odds of having chosen generalist vs specialist practice.

### Study Limitations

This was a cohort study rather than a randomised controlled trial and therefore has not unequivocally established a causal link between medical student origins in a low SES address at entry to medical school and the eventual decision to practice in an a low SES area. The use of the AHPRA data-base to assess current practice location may have failed to capture shorter periods of practice in underserved areas either before or during 2016 and probably under-estimates ultimate practice in either a lower SES or outer metropolitan area. The use of either an individual’s postcode or the postcode of the current location of medical practice as a surrogate for socio-economic status imputes an index (IRSAD) for all people living in that defined area and may not be truly reflective of socio-economic status for each individual in that area [24]. The study was confined to a single medical school and the results may not be generalizable to other Australian medical schools or international institutions.

## Conclusions

This study has demonstrated a highly significant association between imputed socio-economic status at entry to medical school and ultimate practice in an area of relative socio-economic disadvantage after graduation. In those whose address was in the lower 8 deciles for socio-economic disadvantage at medical school entry, a 2-fold increase was seen in the odds of currently practicing in communities in the 8 lower deciles for socio-economic disadvantage. This remained highly significant but reduced to a 1.63 fold increase when all other possible predictors were also taken into account. We conclude that widening participation in medical school to students from more diverse socio-educational backgrounds is likely to increase the distribution of the medical workforce to ultimate service across areas representative of a broader socio-economic spectrum.

## Additional file

Additional file 1:
**Table S1 and Table S2.** **Table S1.** Multivariate logistic regression with low (1^st^ to 8^th^) vs high (9^th^ to 10^th^) IRSAD decile of current practice as the dependent variable and selection and socio-demographic factors as the predictor variables - rural background graduates excluded (*N* = 2638) (Nagelkerke R Square = 0.104). **Table S2**. Multivariate logistic regression with inner vs outer metropolitan address of current practice as the dependent variable and selection and socio-demographic factors as the predictor variables - city based graduates only (*N* = 2473) (Nagelkerke R Square = 0.046). (DOCX 24 kb).

## Abbreviations

AHPRA: Australian Health Practitioner Regulation Agency
ATAR: Australian Tertiary Admissions Rank
BMP: Bonded Medical Places Scheme
CI: Confidence intervals
DWS: District of Workforce Shortage
GAMSAT: Graduate Australian Medical School Admission Test
GPA: Grade point average
IRSAD: Index of Relative Socioeconomic Advantage and Disadvantage
MRBS: Medical Rural Bonded Scholarship Scheme
OR: Odds ratio
SEIFA: Socio-Economic Indices for Areas
SES: Socio-economic status
UMAT: Undergraduate Medicine and Health Sciences Admission Test
UWA: The University of Western Australia

## References

[1] Wittern-Sterzel R (2003). “Politics is nothing else than large scale medicine”--Rudolf Virchow and his role in the development of social medicine. Verh Dtsch Ges Pathol.

[2] Dussault G, Franceschini MC (2006). Not enough there, too many here: understanding geographical imbalances in the distribution of the health workforce. Hum Resour Health.

[3] Larkins S, Evans R (2014). Greater support for generalism in rural and regional Australia. Aust Fam Physician.

[4] Armstrong K, Tess D, Walsh J, Hui Q, Lam W, Brookes R, et al. Technical paper: general practice workforce modelling. 2005. Australian Medical Workforce Advisory Committee (AMWAC) & AMWAC General Practice Working Party.

[5] Hyndman JC, Holman CD (2001). Accessibility and spatial distribution of general practice services in an Australian city by levels of social disadvantage. Soc Sci Med.

[6] Roeger LS, Reed RL, Smith BP (2010). Equity of access in the spatial distribution of GPs within an Australian metropolitan city. Aust J Prim Health.

[7] Rabinowitz HK, Diamond JJ, Veloski JJ, Gayle JA (2000). The impact of multiple predictors on generalist physicians’ care of underserved populations. Am J Public Health.

[8] Wayne SJ, Kalishman S, Jerabek RN, Timm C, Cosgrove E (2010). Early predictors of physicians’ practice in medically underserved communities: a 12-year follow-up study of university of New Mexico school of medicine graduates. Acad Med.

[9] Odom WK, Ryan G, Ramey R, Nunez FL, Beltran R, Splawn RG (2010). Recruiting and retaining primary care physicians in urban underserved communities: the importance of having a mission to serve. Am J Public Health.

[10] Avery DM, Wheat JR, Leeper JD, McKnight JT, Ballard BG, Chen J (2012). Admission factors predicting family medicine specialty choice: a literature review and exploratory study among students in the rural medical scholars program. J Rural Health.

[11] Murray RB, Larkins S, Russell H, Ewen S, Prideaux D (2012). Medical schools as agents of change: socially accountable medical education. Med J Aust.

[12] Larkins S, Michielsen K, Iputo J, Elsanousi S, Mammen M, Graves L (2015). Impact of selection strategies on representation of underserved populations and intention to practise: international findings. Med Educ.

[13] Worley P, Martin A, Prideaux D, Woodman R, Worley E, Lowe M (2008). Vocational career paths of graduate entry medical students at flinders university: a comparison of rural, remote and tertiary tracks. Med J Aust.

[14] Playford DE, Evans SF, Atkinson DN, Auret KA, Riley GJ (2014). Impact of the rural clinical school of western Australia on work location of medical graduates. Med J Aust.

[15] McDonnel SA, Lowe MP (2008). Efficiency of clinical training at the northern territory clinical school: placement length and rate of return for internship. Med J Aust.

[16] Eley DS, Synnott R, Baker PG, Chater AB (2012). A decade of Australian rural clinical school graduates--where are they and why?. Rural Remote Health.

[17] Kondalsamy-Chennakesavan S, Eley DS, Ranmuthugala G, Chater AB, Toombs MR, Darshan D (2015). Determinants of rural practice: positive interaction between rural background and rural undergraduate training. Med J Aust.

[18] Jolly P. Diversity of US medical students by parental income. Analysis in Brief. 2008. Association of American Medical Colleges Washington, D.C. Available from: https://www.aamc.org/download/102338/data/. Accessed 16 Dec 2016.

[19] 19. BMA Equal Opportunities Committee. BMA, Equality and Diversity Committee. Equality and diversity in UK medical schools. 2009. Available from: http://www.nhshistory.net/bmastudentreport2009.pdf. Accessed 16 Dec 2016.

[20] Hunt M, Pywell S, Le L, Lay D. UMAT2012. Report on the 2012 Undergraduate Medicine and Health Sciences Admission Test. 2012. Australian Council for Education Research.

[21] Pywell S, Hunt M, Edwards J, Le L, Nguyen V. Report - Graduate Australian Medical School Admission Test 2012. 2012. Australian Council for Education Research.

[22] The Australian Burea of Statistics. The Australian Standard Geographical Classification (ASGC) Remoteness Structure. 2011. Available from: http://www.abs.gov.au/websitedbs/d3310114.nsf/home/remoteness+structure. Accessed 16 Dec 2016.

[23] Australian Standard Classification of Countries for Social Statistics, Second Edition. 2008. Australian Bureau of Statistics, Catalogue No. 1269.0. Available from: http://www.abs.gov.au/ausstats/abs@.nsf/Lookup/1269.0main+features102011. Accessed 16 Dec 2016.

[24] 24. Australian Bureau of Statistics. 2039.0 - Information Paper: An Introduction to Socio-Economic Indexes for Areas (SEIFA). 2006. Available from: http://www.abs.gov.au/AUSSTATS/abs@.nsf/Lookup/2039.0Main+Features12006?OpenDocument. Accessed 16 Dec 2016.

[25] Puddey IB, Mercer A (2013). Socio-economic predictors of performance in the undergraduate medicine and health sciences admission test (UMAT). BMC Med Educ.

[26] Australian Government Department of Health. Doctor Connect. 2016. http://www.doctorconnect.gov.au/locator. Accessed 16 Dec 2016.

[27] Department of Health and Ageing. More Doctors for Outer Metropolitan Areas - relocation incentive grant program guidelines. 2007. Commonwealth of Australia.

[28] Puddey IB, Mercer A, Carr SE, Louden W. Potential influence of selection criteria on the demographic composition of students in an Australian medical school. BMC Med Educ. 2011;11. 10.1186/1472-6920-11-97 PMC3233506 22111521

[29] Shipman SA, Jones KC, Erikson CE, Sandberg SF (2013). Exploring the workforce implications of a decade of medical school expansion: variations in medical school growth and changes in student characteristics and career plans. Acad Med.

[30] Orlowski J, Jones K, Whatley M. Results of the 2015 Medical School Enrollment Survey. Washington: Association of American Medical Colleges; 2016. Available from: https://www.aamc.org/download/102338/data/. Accessed 16 Dec 2016.

[31] James D, Yates J, Nicholson S. Comparison of a level and UKCAT performance in students applying to UK medical and dental schools in 2006: cohort study. Br Med J. 2010;340. 10.1136/bmj.c478 PMC2824099 20160316

[32] Reiter HI, Lockyer J, Ziola B, Courneya CA, Eva K (2012). Should efforts in favor of medical student diversity be focused during admissions or farther upstream?. Acad Med.

[33] Mitchell CJ, Shulruf B, Poole PJ (2010). Relationship between decile score of secondary school, the size of town of origin and career intentions of New Zealand medical students. J Prim Health Care.

[34] Gore J, Holmes K, Smith M, Southgate E, Albright J (2015). Socioeconomic status and the career aspirations of Australian school students: testing enduring assumptions. Aust Educ Res.

[35] Thompson MN, Subich LM (2006). The relation of social status to the career decision-making process. J Vocat Behav.

[36] Ward AM, Kamien M, Lopez DG (2004). Medical career choice and practice location: early factors predicting course completion, career choice and practice location. Med Educ.

