# Supplementary material for: Impact of medical student origins on the likelihood of ultimately practicing in areas of low vs high socio-economic status
Source: BMC Med Educ. 2017 Jan 5;17:1. doi: 10.1186/s12909-016-0842-7 (PMC5215143; doi:10.1186/s12909-016-0842-7)
Supplement: Additional file 1: — Table S1 and Table S2. Table S1. Multivariate logistic regression with low (1st to 8th) vs high (9th to 10th) IRSAD decile of current practice as the dependent variable and selection and socio-demographic factors as the predictor variables - rural background graduates excluded (N = 2638) (Nagelkerke R Square = 0.104). Table S2. Multivariate logistic regression with inner vs outer metropolitan address of current practice as the dependent variable and selection and socio-demographic factors as the predictor variables - city based graduates only (N = 2473) (Nagelkerke R Square = 0.046). (DOCX 24 kb). [file 12909_2016_842_MOESM1_ESM.docx]

**Supplementary Table 1** Multivariate logistic regression with low (1^st^ to 8^th^) vs high (9^th^ to 10^th^) IRSAD decile of current practice as the dependent variable and selection and socio-demographic factors as the predictor variables - rural background graduates excluded (N=2638) (Nagelkerke R Square = 0.104).

| Predictor Variable | B | S.E. | P Value | Odds Ratio (95% CI) |
| --- | --- | --- | --- | --- |
|  |  |  |  |  |
| **Quota-based entry pathway** |  |  |  |  |
| No quota-based entry |  |  |  | 1.0 |
| Quota-based entry | 0.671 | 0.461 | 0.145 | 1.96 (0.79, 4.83) |
| **Selection factors** |  |  |  |  |
| ATAR or GPA alone |  |  |  | 1.0 |
| ATAR or GPA, UMAT or GAMSAT and  Interview score | -0.151 | 0.130 | 0.243 | 0.86 (0.67, 1.11) |
| **Medical Rural Bonded Scholarship** |  |  |  |  |
| No Medical Rural Bonded Scholarship |  |  |  | 1.0 |
| Medical Rural Bonded Scholarship | 0.811 | 0.369 | **0.028** | 2.25 (1.09, 4.63) |
| **Bonded Medical Place** |  |  |  |  |
| No bonded medical place |  |  |  | 1.0 |
| Bonded medical place | -0.114 | 0.315 | 0.716 | 0.89 (0.48, 1.65) |
| **Age at Completion** |  |  |  |  |
| 24 yr or younger |  |  |  | 1.0 |
| 25 yr or older | 0.217 | 0.110 | **0.049** | 1.24 (1.00, 1.54) |
| **Sex** |  |  |  |  |
| Male |  |  |  | 1.0 |
| Female | 0.024 | 0.093 | 0.796 | 1.02 (0.85, 1.23) |
| **School Type** |  |  |  |  |
| Government |  |  |  | 1.0 |
| Independent | -0.195 | 0.096 | **0.042** | 0.82 (0.68, 0.99) |
| **Country of Origin** |  |  |  |  |
| Oceania |  |  |  | 1.0 |
| Other | -0.164 | 0.096 | 0.086 | 0.85 (0.70, 1.02) |
| **Registration Type** |  |  |  |  |
| Specialist |  |  |  | 1.0 |
| General registration | 0.637 | 0.141 | **<0.001** | 1.89 (1.43, 2.50) |
| General Practice | 1.318 | 0.118 | **<0.001** | 3.74 (2.96, 4.71) |
| **IRSAD Decile of Address at Entry** |  |  |  |  |
| Deciles 1-8 | 0.425 | 0.105 | **<0.001** | 1.53 (1.25, 1.88) |
| Deciles 9-10 |  |  |  | 1.0 |

Significant P values are in bold-face type

**Supplementary Table 2** Multivariate logistic regression with inner vs outer metropolitan address of current practice as the dependent variable and selection and socio-demographic factors as the predictor variables - city based graduates only (N=2473) (Nagelkerke R Square = 0.046).

| Predictor Variable | B | S.E. | P Value | Odds Ratio (95% CI) |
| --- | --- | --- | --- | --- |
|  |  |  |  |  |
| **Quota-based entry pathway** |  |  |  |  |
| No quota-based entry |  |  |  | 1.0 |
| Quota-based entry | 0.345 | 0.241 | 0.152 | 1.41 (0.88, 2.27) |
| **Selection factors** |  |  |  |  |
| ATAR or GPA alone |  |  |  | 1.0 |
| ATAR or GPA, UMAT or GAMSAT and  Interview score | -0.269 | 0.169 | 0.111 | 0.76 (0.55, 1.06) |
| **Medical Rural Bonded Scholarship** |  |  |  |  |
| No Medical Rural Bonded Scholarship |  |  |  | 1.0 |
| Medical Rural Bonded Scholarship | 0.576 | 0.435 | 0.185 | 1.78 (0.76, 4.17) |
| **Bonded Medical Place** |  |  |  |  |
| No bonded medical place |  |  |  |  |
| Bonded medical place |  |  |  |  |
| **Age at Completion** |  |  |  |  |
| 24 yr or younger |  |  |  | 1.0 |
| 25 yr or older | 0.080 | 0.140 | 0.566 | 1.08 (0.82, 1.43) |
| **Sex** |  |  |  |  |
| Male |  |  |  | 1.0 |
| Female | -0.023 | 0.118 | 0.848 | 0.97 (0.78, 1.23) |
| **School Type** |  |  |  |  |
| Government |  |  |  | 1.0 |
| Independent | -0.139 | 0.121 | 0.251 | 0.87 (0.69, 1.10) |
| **Country of Origin** |  |  |  |  |
| Oceania |  |  |  | 1.0 |
| Other | -0.177 | 0.123 | 0.150 | 0.84 (0.66, 1.07) |
| **Registration Type** |  |  |  |  |
| Specialist |  |  |  | 1.0 |
| General registration | 0.624 | 0.180 | **0.001** | 1.87 (1.31, 2.66) |
| General Practice | 0.823 | 0.155 | **<0.001** | 2.28 (1.68, 3.09) |
| **IRSAD Decile of Address at Entry** |  |  |  |  |
| Deciles 1-8 | 0.418 | 0.131 | **0.001** | 1.52 (1.17, 1.97) |
| Deciles 9-10 |  |  |  | 1.0 |

Significant P values are in bold-face type
